# ERK: A Key Player in the Pathophysiology of Cardiac Hypertrophy

**DOI:** 10.3390/ijms20092164

**Published:** 2019-05-01

**Authors:** Simona Gallo, Annapia Vitacolonna, Alessandro Bonzano, Paolo Comoglio, Tiziana Crepaldi

**Affiliations:** 1 Candiolo Cancer Institute, FPO-IRCCS, 10060 Candiolo (TO), Italy; simona.gallo@ircc.it (S.G.); annapia.vitacolonna@ircc.it (A.V.); alessandro.bonzano@ircc.it (A.B.); pcomoglio@gmail.com (P.C.); 2 Department of Oncology, University of Turin, 10143 Orbassano (TO), Italy

**Keywords:** ERK pathway, adaptive and maladaptive hypertrophy, anthracycline-induced cardiotoxicity, hypertrophic cardiomyopathy, RASopathies, target therapies

## Abstract

Cardiac hypertrophy is an adaptive and compensatory mechanism preserving cardiac output during detrimental stimuli. Nevertheless, long-term stimuli incite chronic hypertrophy and may lead to heart failure. In this review, we analyze the recent literature regarding the role of ERK (extracellular signal-regulated kinase) activity in cardiac hypertrophy. ERK signaling produces beneficial effects during the early phase of chronic pressure overload in response to G protein-coupled receptors (GPCRs) and integrin stimulation. These functions comprise (i) adaptive concentric hypertrophy and (ii) cell death prevention. On the other hand, ERK participates in maladaptive hypertrophy during hypertension and chemotherapy-mediated cardiac side effects. Specific ERK-associated scaffold proteins are implicated in either cardioprotective or detrimental hypertrophic functions. Interestingly, ERK phosphorylated at threonine 188 and activated ERK5 (the big MAPK 1) are associated with pathological forms of hypertrophy. Finally, we examine the connection between ERK activation and hypertrophy in (i) transgenic mice overexpressing constitutively activated RTKs (receptor tyrosine kinases), (ii) animal models with mutated sarcomeric proteins characteristic of inherited hypertrophic cardiomyopathies (HCMs), and (iii) mice reproducing syndromic genetic RASopathies. Overall, the scientific literature suggests that during cardiac hypertrophy, ERK could be a “good” player to be stimulated or a “bad” actor to be mitigated, depending on the pathophysiological context.

## 1. Introduction

The mitogen-activated protein kinase (MAPK) pathway (also known as the RAS-RAF-MEK-ERK pathway) is a central signaling cascade activated by receptor tyrosine kinases (RTKs) upon binding by extracellular mitogenic ligands [1,2]. RAS is a small GTP-binding protein that is activated by tyrosine kinase receptors and transmits the signal from the cell membrane to the nucleus. At the plasmamembrane, RAS activates the RAF kinase (MAPKKK), which in turn activates the MEK kinase (MAPKK), which consecutively stimulates the ERK (extracellular signal-regulated kinase; MAPK) through serial phosphorylation. The prototypical ERK 1/2 isoforms (here named ERK in its singular noun) are responsive to stimulation to growth factors and have apparent redundant functions. Once activated, ERK translocates to the nucleus and phosphorylates multiple substrates, including transcription factors, such as CREB and Elk1. The activation and repression of nuclear targets result in the induction of growth and proliferation and in the prevention of cell death [1]. Moreover, ERK phosphorylates intracellular substrates in the cytoplasm, among which cytoskeletal and adherens junction proteins as well as apoptotic and cell cycle regulators stand out. Since proliferation and cell growth are important processes for heart development, it is not surprising that ERK plays a central role in cardiac physiology [3]. Importantly, the ERK molecules are implicated in several forms of cardiac hypertrophy and progression to heart failure [3,4,5,6,7,8]. The role of ERK in the hypertrophic process is, however, controversial and has not been fully understood [9]. ERK seems to be involved in the induction of the adaptive hypertrophy, since transgenic mice expressing activated MEK1, the specific activator of ERK, show concentric cardiac hypertrophy with enhanced contractile force [4]. Notably, these mice did not show signs of pathological hypertrophy, such as fibrosis or sudden death. On the other hand, the overexpression of activated MEK5, which specifically stimulates ERK5 (the big MAPK), facilitates maladaptive, eccentric cardiac hypertrophy and leads to cardiomyopathy and sudden death [10]. Not only the specificity, but also the intensity, duration, and localization of ERK signaling may be directed towards adaptive or maladaptive hypertrophy. The balance of ERK signaling decides its beneficial/detrimental role in the hypertrophic process. 

In this review, we examine the more recent literature regarding the function of ERK pathway in adaptive or maladaptive remodeling involved in cardiac hypertrophy. We focus on the actions mediated by ERK in cardiomyocytes, which represent the major cell type of the cardiac organ. Although ERK signaling has an impact also on fibroblasts and endothelial cells, such discussion is beyond the scope of this review. Moreover, we address the involvement of ERK signaling in hereditary cardiomyopathies and RASopathies, in which hypertrophy is a typical pathological feature. Finally, we discuss recent scientific results addressing ERK signaling as a therapeutic target to manage cardiac hypertrophy.

## 2. Overview of Cardiac Hypertrophy

**Figure 1:**
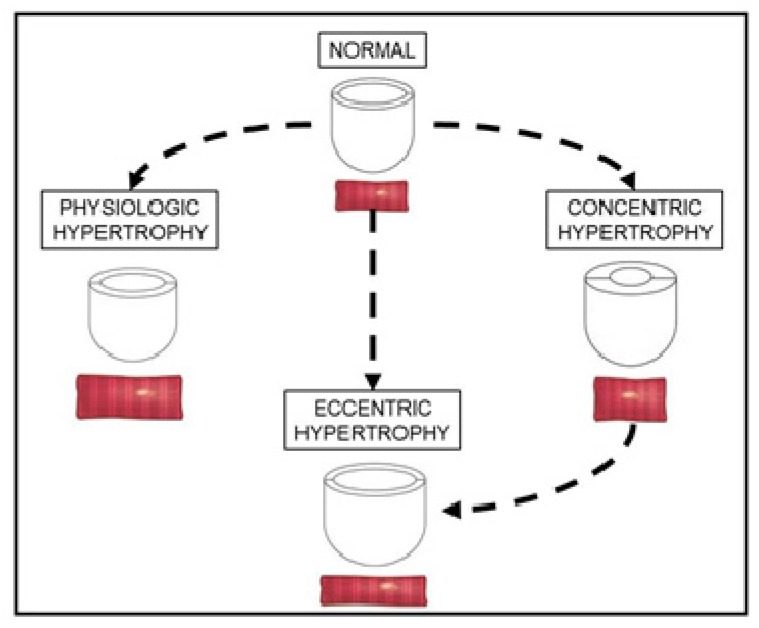
Simplified view of cardiac hypertrophy. The normal heart develops left ventricular remodeling in response to physiological (exercise and pregnancy) and pathological (pressure or volume overload, myocardial infarction, hypertension, drug toxicity, and congenital heart defects) stimuli. In the physiological hypertrophy, cardiomyocytes increase in length and width. In the concentric hypertrophy, cardiomyocytes mostly increase in width compared with length. In the eccentric hypertrophy, cardiomyocytes mostly grow in length compared with width, leading to dilated cardiomyopathy. Except for physiological hypertrophy, hypertrophic remodeling can progress to contractile dysfunction and heart failure.

The heart reacts to a large number of physiological and pathological stimuli through cardiac hypertrophy [11]. Since the cardiac muscle cells are terminally differentiated and have a limited ability to proliferate, the heart modifies its volume and muscle mass by hypertrophic remodeling to increase the contractile force and workload. During this dynamic response, the cardiomyocytes increase in size, change their shape, modify the gene expression, and remodel the cytoskeleton and the extracellular matrix (ECM) [12]. Importantly, during the postnatal development, hypertrophy is the prevalent way for the heart to grow [13]. At adult age, strong exercise results in physiological hypertrophy typified by wall and septal thickness growth (Figure 1). Stimuli of various origins, such as trophic, mechanical, hemodynamic, and neurohumoral signals, lead to cardiac hypertrophy. In fact, the increase in cardiac muscle mass and force produces beneficial effects aiming to normalize wall stress and preserve cardiac output while blood filling is impaired. Pressure overload usually results in concentric hypertrophy with relative increase in cardiomyocyte width, while volume overload typically produces eccentric hypertrophy with increase in cardiomyocyte length, left ventricle dilatation, and heart failure (Figure 1). Nevertheless, chronic concentric hypertrophy is the first step for the deterioration and failure of the heart, since it leads to changes in gene expression program, contractile dysfunction, and extracellular remodeling [14]. In fact, clinical evidence shows that hypertrophy is an important predictive factor for adverse outcomes and increased cardiovascular mortality due to the development of heart failure, dilated cardiomyopathy, ischaemic heart disease, and sudden death [15]. Pathological hypertrophy is induced by different detrimental processes, such as pressure or volume overload, myocardial infarction, hypertension, drug toxicity, and congenital heart defects. Thus, cardiac hypertrophy is a balanced process: it is adaptive and compensatory when it is moderated and produces beneficial effects for the heart contraction; it becomes maladaptive when it is chronic and opens the way to pathological diseases (Figure 1). For this reason, hypertrophy could be a “good” player to be stimulated or a “bad” mechanism to be prevented depending on the pathophysiological context.

Mechanistically, hypertrophy is a very complex process affecting the cardiomyocyte, in which several regulatory steps are activated in response to cardiac injury. A great number of intracellular pathways have been associated with the hypertrophic response [14]. These pathways are intertwined, generating a complex response that is still not completely clear. Likely, they interact to regulate the balance that defines whether the hypertrophic process is an adaptive or maladaptive mechanism. This concept could have a significant implication for the development of therapeutic approaches to manage the hypertrophic response and direct the process towards a more favorable outcome.

## 3. The Role of ERK in Adaptive Cardiac Hypertrophy

Hypertrophy is considered an adaptive process when it is coupled to an increase in the request of heart performance without the induction of cardiac damage. Adaptive cardiac hypertrophy typically occurs in healthy individuals following exercise or during pregnancy. Moreover, the early-induced hypertrophic response to pathological stimuli is a compensatory mechanism that is used by cardiomyocytes to overcome the elevated workload or injury. However, when the pro-hypertrophic signaling remains sustained and chronic, it becomes maladaptive and induces heart damage. The molecular pathways involved in the pro-hypertrophic response are often intertwined and play roles in both the adaptive and maladaptive hypertrophy. Physiological hypertrophy, such as that associated with swimming or running, is regulated in large part by the growth hormone/IGF axis via signaling through the PI3K/Akt pathway [16]. However, the ERK pathway also participates in the induction of hypertrophy in response to different external pathophysiological stimuli. In this section, we focus on the role exerted by ERK in adaptive hypertrophy.

### 3.1. Adaptive Concentric Hypertrophy

**Figure 2:**
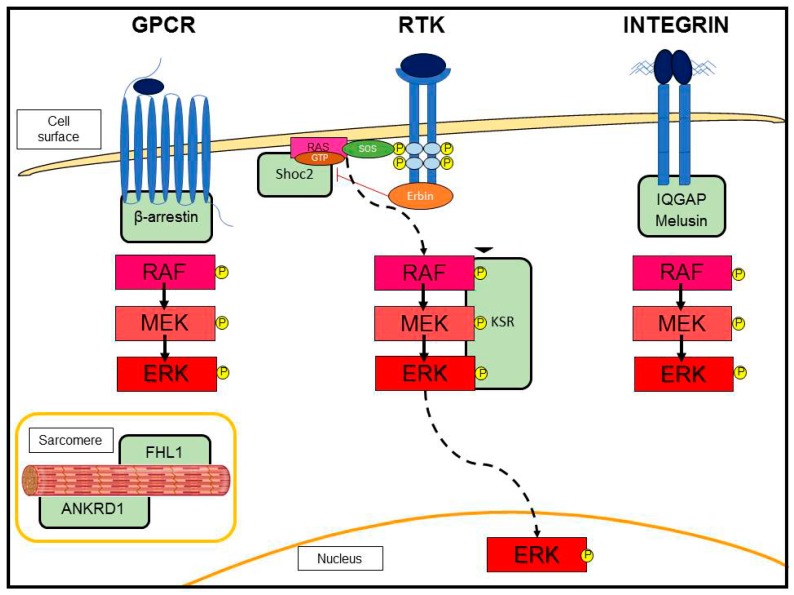
Schematic view of the ERK pathway in response to growth factors, hormones, and mechanical stress. The prototypical activation of ERK cascade is initiated at the plasmamembrane by receptor tyrosine kinases (RTKs) in response to growth factors. Activated RTKs promote RAS stimulation through recruitment of SOS exchange factor. RAS facilitates the activation of MEK-ERK cascade through serial phosphorylation. Once activated, ERK translocates to the nucleus and phosphorylates transcription factors, modulating the transcription of hundreds of genes. In cardiac myocytes under stress (aortic stenosis and hypertension), ERK is activated in response to G protein-coupled receptors (GPCRs), and/or “stretch-sensitive” sensors, such as membrane bound integrins, and the sarcomere. The activation of ERK cascade is regulated by scaffold proteins (KSR, Shoc2, Erbin, β-arrestin, IQGAP, Melusin, FHL1, and ANKRD1), which bind components of the RAF-MEK-ERK module, facilitating their functional interaction and subcellular localization. Scaffolds also link the ERK activation to specific upstream signal and affect the duration of the signal.

Chronic pressure overload is a pathological condition that occurs in patients with hypertension or stenosis of the aortic valve. At the beginning of pressure overload, the heart responds to stress by concentric hypertrophy. When prolonged, this compensatory process may progress to eccentric hypertrophy and culminates in myocardial dysfunction and heart failure. The transverse aortic constriction (TAC) animal model, which mimics the pressure overload human condition, was extensively used to investigate in a spatial and temporal manner the molecular determinants involved in this pathology [17,18]. Importantly, the TAC approach allows one to study the disease progression from adaptive hypertrophy to heart failure, identifying the signaling pathways involved in this transition. TAC mice showed an increase in phosphorylation of ERK in the first phase of pressure overload stimulation, when the contractile function is maintained, whereas P-ERK was decreased at the time of functional decompensation, when fibrosis and cardiomyocytes apoptosis appear. Thus, ERK is stimulated during the early adaptive concentric hypertrophy and its activation is reduced during the late detrimental eccentric hypertrophy [19]. In cardiac myocytes under pressure overload, ERK is activated in response to G protein-coupled receptors (GPCRs) [20,21,22,23,24], and/or “stretch-sensitive” sensors, such as membrane bound integrins [25], and the sarcomere itself [26]. In particular, β-arrestin, which specifically binds GPCRs and blocks downstream G protein activation, mediates the cross-talk of GPCRs with the RAS-RAF-MEK-ERK module (Figure 2) [20,27]. Moreover, β-arrestin facilitates the transactivation of the epidermal growth factor receptor (EGFR) [28,29]. Transgenic mice with cardiac-specific expression of a dominant negative form of RAF-1 (DN-RAF) were sensitized to pressure overload pathological effects. DN-RAF mice subjected to TAC did not develop adaptive hypertrophy and showed an increased death rate. Importantly, the cardiomyocyte apoptosis was enhanced, indicating that the absence of RAF-1 blocks both the hypertrophic and survival signaling [30]. In line with these results, Mutlak et al. [31] showed that the transgenic model of constitutively activated ERK in cardiomyocytes developed adaptive hypertrophy with increased ventricular contractile function under pressure overload. The ERK transgenic mice showed also a strong reduction in fibrosis accumulation [31]. Accordingly, mice overexpressing DUSP6, the specific phosphatase of ERK, were sensitized to heart failure progression when subjected to long-term TAC [32]. Overall, these scientific reports suggest that (i) downregulation of ERK is implicated in the transition from compensated hypertrophy to maladaptive hypertrophic heart failure during pressure overload, and that (ii) ERK is required to prevent eccentric growth in the context of pressure overload. Indeed, stimulation of this pathway could be a therapeutic strategy to maintain the heart performance during this pathological hemodynamic condition.

### 3.2. G-Protein-Coupled Receptor (GPCR)-Induced Adaptive Hypertrophy

Stimulation of GPCRs by agonists (for example, angiotensin II (AngII) and adrenergic receptors) recruits β-arrestin, which acts as a molecular scaffold for the RAF-MEK-ERK pathway (Figure 2). G-protein and β-arrestin signaling pathways are not discussed here (reviewed in Reiter Lefkowitz 2013 [33]). Instead, we focus on studies involving GPCR-activated ERK signaling in cardiac hypertrophy. GPCRs are a conserved family of transmembrane receptors that regulate a vast multitude of physiological and pathological processes in the heart. The two main classes of GPCRs in this organ are the adrenergic and angiotensin receptors. The adrenergic receptors enable the communication between the sympathetic nervous and cardiovascular systems. Their role in the hypertrophic process remains controversial. The β-adrenergic receptor (βAR) is known to be involved in the pathological induction of heart failure. In fact, chronic activation of βAR induces apoptosis and adverse cardiac hypertrophic remodeling [24]. On the other hand, α-adrenergic (αAR) receptors, interacting with each other and with other adrenergic receptors, seem to positively regulate cardiac contractile and hypertrophic responses [24]. Interestingly, data from the literature demonstrate that the α-adrenergic pathway is involved in the development of adaptive hypertrophy during cardiac stress remodeling via the activation of ERK signaling [34,35]. In fact, α 1A/C-AR and α 1B-AR double knockout mouse showed a strong reduction in ERK pathway activation and a small heart with normal contractile function, but reduced cardiac output. Importantly, the absence of α-adrenergic receptors led to an increased mortality rate under pressure overload stimulus [34]. In addition, the loss of α1-adrenergic signaling induced greater pathological remodeling post-myocardial infarction (MI). In fact, α 1A-AR knockout showed a strong shift towards pathological hypertrophy and heart failure after MI. In parallel, these knockout mice presented reduced ERK activity and the induction of apoptosis [35]. The termination of GPCR signaling occurs by the phosphorylation of the activated receptor and recruitment of the adapter protein β-arrestin, a process called “desensitization.” Besides this function, β-arrestin facilitates the activation of ERK cascade and promotes cell survival [22]. Interestingly, β-arrestin counteracts the classical β-adrenergic-induced heart failure [28,36]. Overall, these data indicate that ERK participates in GPCR-induced adaptive hypertrophic response against cardiac stress.

### 3.3. Scaffold Proteins in ERK Signaling

The activation of MAPK cascade depends also on the interaction with scaffold proteins that enable the formation of specific signaling complexes and subcellular localization (Figure 2). Originally identified in yeast and *C. elegans*, scaffold proteins are now recognized to contribute to the specificity of the MEK-ERK pathway in mammalian cells. These proteins facilitate the interaction of signaling components and stimulate a specific signal transduction [37]. ERK-associated scaffolding proteins regulate the functions of the signaling module by coordinating and organizing its spatiotemporal activation in response to different stimuli. KSR is one of the best-characterized mammalian MAPK scaffold proteins [38]. KSR is downstream RAS and critical in recruiting the RAF-MEK-ERK complex to the plasma membrane as well as regulating MAPK signaling strength and duration at this location [39]. However, its role in cardiac hypertrophy has yet to be investigated. Instead, other ERK-associated scaffolding proteins have been shown to play a central role in the maintenance of cardiomyocyte physiology and the induction of adaptive hypertrophy [40]. We have already mentioned β-arrestin as an important scaffold for ERK activation in response to GPCRs (see above). In addition, Erbin (ErbB2 interacting protein) is able to form a complex with HER2/neu (ErbB2) and β2AR [41]. Exaggerated pathological cardiac hypertrophy was observed in *Erbin*−/− mice. TAC in these mice resulted in rapid decompensation and heart failure [42]. Recently, it has been suggested that Erbin uncouples the Shoc2 scaffold from the RAS-RAF-MEK-ERK pathway, thus behaving as a negative regulator of ERK signaling [43]. Moreover, the ERK-associated scaffolding protein IQGAP1 (IQ motif-containing GTPase-activating protein 1) emerged for its function in cardiac pathophysiology. This protein has many binding partners, which have essential roles in control of cell adhesion and actin cytoskeleton. It connects the integrin signaling with ERK activation, by acting as a scaffold protein for the RAF-MEK-ERK complex. IQGAP1-null mice subjected to TAC display a normal compensatory hypertrophy in the early phase of the stimulus, but after prolonged pressure overload they show acceleration towards the development of eccentric maladaptive hypertrophy leading to decreased heart contractility [44]. This impaired response was due to a blunted activation of the hypertrophic program and an increase in cardiomyocyte apoptosis. Mechanistically, the lack of IQGAP1 prevents the late stimulation of ERK signaling, which is involved in the induction of the adaptive hypertrophy, protection from apoptosis, and the prevention of eccentric growth. Another scaffold protein organizing the ERK cascade in cardiomyocytes and involved in the induction of cardiac compensatory hypertrophy is Melusin [45]. Melusin belongs to the family of heat shock protein 90 machinery, is specifically expressed in skeletal and heart muscles, and binds integrin stretch-sensitive sensors. Melusin null mice did not show defects in heart functions during physiological conditions. On the other hand, these mice failed to activate the adaptive hypertrophic program developing impaired heart contractility in the TAC model [46]. In parallel, mice overexpressing Melusin under cardiac-specific promoter showed improved contractile function and concentric compensatory hypertrophy when the heart was subjected to a prolonged aortic stenosis. In addition to the adaptive hypertrophy, these mice presented reduced inflammation, fibrosis, and apoptosis [47]. Further ERK-associated scaffolding proteins involved in the regulation of hypertrophy are FHL1 (four and a half LIM domain protein-1) and ANKRD1 (ankyrin repeat domain 1). These proteins are myofilament-associated proteins highly induced during the hypertrophic process in mice and humans and are components of the sarcomere-associated biomechanical sensors. Loss of function studies highlighted that both proteins, FHL1 and ANKRD1, have detrimental roles in Gαq and phenylephrine-induced cardiac hypertrophy [40]. *Fhl1*−/− hearts displayed a blunted response to pathological hypertrophy [48]. FHL1 directly interacts with RAF-MEK-ERK to physically insulate the MAPK pathway to the titin N2B region of the sarcomere in a Gq stimulus-specific manner and efficiently transmit MAPK signals to regulate cardiac hypertrophy. *Ankrd1*−/− mice subjected to pressure overload via TAC were also able to maintain a normal cardiac hypertrophic response, similar to control mice [49]. In this case, ANKRD1 transmits MAPK signals to regulate the α-adrenergic associated hypertrophic pathway [50,51]. Altogether, these findings suggest that distinct scaffolds and subcellular localization may modulate ERK signaling in pathological states associated with cardiac hypertrophy.

### 3.4. Cell Death Prevention

The adaptive hypertrophic response is characterized by cell death prevention. In fact, apoptosis is implicated as an important contributor to the pathogenesis of cardiac hypertrophy and heart failure. As mentioned above, the ERK pathway exerts a strong anti-apoptotic function in numerous contexts of cardiac stress. In fact, ERK cascade promotes cardiomyocyte survival in response to pressure overload [19,30], MI [35], and oxidative stress [52]. Mechanistically, the precise biochemical mechanisms for the ERK-mediated anti-apoptotic effect have yet to be defined, although some associations with the Bcl-2 family have been shown in cardiomyocytes [53,54]. In other cell types, ERK prevents apoptosis by various mechanisms. Inhibition of Caspase 8/Bid cleavage [55] and inactivation of the pro-apoptotic Bad [56] and Bim [57] are downstream to ERK. ERK also interferes with mitochondria pro-apoptotic activity through the phosphorylation of caspase-9 at threonine 125 [58]. In addition to suppressing pro-apoptotic proteins, ERK promotes anti-apoptotic molecules, among which are Mcl-1, an anti-apoptotic member of the Bcl-2 family [59], and IEX-1, a target gene of NF-κB (nuclear factor kappa B) that prevents the release of cytochrome c from mitochondria [60]. Whether the same mechanisms contribute to ERK-mediated cardioprotection have yet to be identified.

## 4. The Role of ERK in Maladaptive Cardiac Hypertrophy

The maladaptive hypertrophy is a response to various intrinsic and extrinsic stimuli that rapidly lead to the reduction of cardiac contractility and heart failure. In contrast to the compensatory form, maladaptive hypertrophy is not reversible when the pathological stimulus is removed or corrected. Often the compensatory hypertrophy, whether subjected to continuous stress, results inevitably in decompensation and development of dilated cardiomyopathy. The detrimental hypertrophic process characterizes different cardiovascular diseases, such as hypertension, aortic valve stenosis, or chronic adrenergic receptor stimulation. Although the ERK pathway was identified exerting important functions to develop the adaptive hypertrophy (see Section 3), some scientific data demonstrate the involvement of ERK cascade in the maladaptive hypertrophy. In this section, we focus on the ERK-mediated effects in the harmful hypertrophic process.

### 4.1. Hypertension

Continuous hemodynamic overload induces an unfavorable hypertrophic response. The main pathway involved in hypertension is the renin–angiotensin system. Its main effector hormone is AngII, which induces physiological vasoconstriction, blood pressure regulation, and production of extracellular matrix proteins. In addition, AngII stimulates aberrant cardiac cell growth, as suggested by the high levels of AngII after myocardial infarction and during cardiac hypertrophy [24]. The angiotensin receptors belong to the GPCRs family, in particular the most involved in hypertension is the Gq-coupled AT1R. Many studies support the observation that AngII induces the hypertrophic response on myocardial cells [61]. AngII-mediated detrimental effects have a crucial role in various cardiac diseases, such as inflammation, atherosclerosis, hypertension, and congestive heart failure. Heart failure patients treated with inhibitors of the angiotensin system showed reduced cardiac hypertrophy and slower progression towards heart failure [24]. These hypertrophic effects are mediated by AT1Rs, as demonstrated by the prevention of hypertrophy through AT1R antagonists [62]. In addition, experimental studies demonstrated increased levels of AT1R in the hypertrophic process [63]. Animal models of AT1Rs cardiac-specific overexpression showed enhanced hypertrophy leading to heart failure, in response to pressure overload [64]. On the other hand, the cardiac knockout of AT1Rs presented improved systolic function with attenuation of AngII-mediated hypertrophic response in MI [65]. Importantly, ERK is activated by AT1Rs and participates in the pathological hypertrophic response [61]. In addition, it has been demonstrated that, during hypertension, ERK-induced hypertrophy can be stimulated by mechanical stretch in an AngII-dependent or independent way [66,67]. However, the specific mechanism by which AngII induces ERK-mediated hypertrophic process remains elusive. Recent data show that, once activated by AT1R, P-ERK stabilized the insulin-like growth factor II receptor (IGF-IIR) protein through the HSF1 (heat shock factor 1)/GSK3 (glycogen synthase kinase III) pathway [68]. This suggests a feed forward mechanism involving IGF-IIR in pathological hypertrophic response. Interestingly, mice with cardiac-specific overexpression of AT1R that are unable to couple with G protein presented enhanced hypertrophy with decreased cell death after chronic treatment with AngII. Importantly, this cardioprotective hypertrophy was mediated by the ERK pathway [69]. In a way similar to the βAR system, the AngII receptors are regulated by a “desensitization” mechanism mediated by β-arrestin (see above). Besides its desensitization role, β-arrestin mediates the transactivation of EGFR, stimulating ERK and thus inducing cardioprotection against AngII-mediated heart failure [36]. Thus, recruitment, activation, and scaffolding of other cytoplasmic signaling complexes may modulate and steer ERK signaling towards adaptive or maladaptive hypertrophy.

### 4.2. Anthracycline-Induced Cardiotoxicity (CTX)

Anti-cancer therapy may lead to cardiac side effects with a great impact on the efficacy of treatment and on the patient’s quality of life. Among chemotherapeutic agents, anthracyclines emerge for their aggressive anti-tumoral activity but also for their strong detrimental effects on the heart. For this reason, their clinical application is limited. Some scientific reports associated anthracycline-CTX to the development of maladaptive hypertrophy, resulting in heart failure [70]. Interestingly, the ERK pathway seems to be involved in anthracycline-CTX, but its role in this pathological context has yet to be elucidated. A number of reports showed that ERK is stimulated by doxorubicin (doxo), likely via mitochondrial ROS (reactive oxygen species) release [71,72,73]. In line with this, our own unpublished data confirm that P-ERK is strongly induced by doxo in vitro in a dose- and time-dependent manner. Mechanistically, ERK participates to doxo-CTX by stimulating a heat shock factor, HSF2, which activates the AngII receptor AT1R. AT1R induction leads to cardiac cell damage and heart failure [71]. In cardiomyocytes, doxo-induced apoptosis was prevented by the pharmacological inhibition of ERK, suggesting that ERK signaling may exert a pro-apoptotic function in the setting of doxo-CTX [72]. However, the mechanisms by which the ERK pathway induces apoptosis in cardiomyocytes need further investigation. On the other hand, some scientific literature supports the cardioprotective action mediated by ERK cascade against anthracycline-CTX [74,75]. Likely, ERK activation exerts both detrimental and cardioprotective functions, and the final outcome depends on the localization, intensity, and duration of the signal. Lou et al. suggest that ERK-mediated beneficial effects against doxo-CTX derive from a sustained activation of ERK, since a transient and short stimulation fails to prevent heart failure [76]. In line with this, our preliminary data show that the ERK pathway exerts an adaptive response to anthracycline-CTX; in fact, the treatment with the ERK inhibitor sensitizes cardiomyocytes to doxo-mediated pro-apoptotic and genotoxic effects. The combination of doxo with trastuzumab, the humanized monoclonal antibody against the erythroblastic leukemia viral oncogene homolog 2 (ErbB2), also known as epidermal growth factor receptor-2 (HER2), demonstrated a significant reduction in morbidity and mortality in breast cancer patients. However, severe cardiotoxic effects emerged when trastuzumab was administered together with doxo [77]. Trastuzumab administered alone resulted in a safe cardiac profile [78]. It is likely that the HER2-mediated pathway is required as a cardioprotective mechanism in response to anthracycline treatment and the blockage of this pathway exacerbates the doxo-derived CTX [79]. Indeed, HER2 signaling protects cardiomyocytes from anthracycline-induced apoptosis and calcium/ mitochondrial dysregulation [80,81,82]. HER2 receptor is known to activate the prosurvival ERK signaling in cardiomyocytes [83]. Thus, interfering with HER2-mediated ERK signaling during or immediately after anthracycline treatment could contribute to increase cardiomyocyte damage. Accordingly, lapatinib, a dual tyrosine kinase inhibitor targeting the HER2 and EGFR pathways, which leads to CTX when co-administered with doxo, decreases the activation of ERK [84]. Recently, Mohan et al. showed that trastuzumab dysregulates HER2 activity, leading to the activation of ERK/mTOR signaling and the inhibition of autophagy in human primary cardiomyocytes [85]. Thus, the function of ERK in the mechanism of trastuzumab-mediated CTX in the context of doxo is complex and has yet to be completely understood.

### 4.3. ERK Phosphorylation at Threonine 188 (T188)

This new autophosphorylation site was discovered by Lorenz et al. in 2009 [86]. When phosphorylated on this specific site, ERK induces maladaptive hypertrophy, as demonstrated in hypertrophic cardiomyocytes, animal models subjected to pressure overload, and failing human hearts [86]. In addition, mice dominant-negative for ERK-T188 showed attenuation of pathological hypertrophy in response to phenylephrine, an agonist for the adrenergic receptors, and chronic pressure overload. Importantly, the lack of ERK-T188 did not produce any effect on the induction of the adaptive hypertrophy and cell survival mediated by ERK. Moreover, phosphorylation on ERK-T188 was found with strong prevalence in patients with a rapid progressing course of aortic valve stenosis [87]. As cited above, the β-adrenergic receptor pathway is associated with an induction of hypertrophy leading to failing heart [24,88]. In particular, β1AR is the subtype with a major role in the induction of pathological hypertrophy, fibrosis, and heart failure. Notably, it has been demonstrated that β1AR exerts its hypertrophic function by induction of ERK phosphorylation at T188. Mechanistically, phosphorylation at T188 is produced by direct interaction between the Gs-derived Gβγ subunits of the receptor and the activated ERK [88]. Overall, these data indicate this additional phosphorylation as a promising pharmacological target to manage the detrimental hypertrophic process without interfering with the adaptive ERK-mediated hypertrophy [89].

### 4.4. ERK5

Also known as big MAPK 1 (BMK1), ERK5 needs a separate and specific discussion. It is characterized by a specific activating MAPKK (MEK5) and is activated by both RTKs and GPCRs [90]. ERK5 shows a different role in regulation of the hypertrophic process respect to ERK. In fact, experiments performed in vitro in cardiomyocytes with constitutively active and dominant-negative forms of MEK5 demonstrate that ERK5 is required for elongation and serial assembly of sarcomeres. Furthermore, MEK5 transgenic mice develop eccentric cardiac hypertrophy that leads to heart failure [10]. Scientific data demonstrate that ERK5 is involved in the development of detrimental hypertrophy in different pathological systems: (i) mice with cardiomyocyte-specific deletion of ERK5 showed reduction in hypertrophic response and induction of apoptosis during pressure overload [91]; (ii) stimulation of ERK5 was associated with eccentric and detrimental hypertrophy in an animal model of long-term intermittent cardiac hypoxia [92]; (iii) ERK5 activation is promoted by AngII hypertrophic detrimental stimulus [93]. In contrast, Cameron et al. found that the cardiac specific overexpression of the α isoform of MEK5 leads to enhanced contractile function and decreased cardiomyocyte apoptosis after ischaemic cardiac damage [94]. The reason for these discrepancies with the transgenic mice of Nicol et al. [10] is not clear, one of the causes could be the different MEK5 isoforms used in the two studies. In addition, Chen et al. found that, in a model of anthracycline-CTX, the induction of ERK5 by a mix of anti-oxidant molecules plays a cardioprotective action avoiding the development of heart failure [95]. Although the role of ERK5 in heart diseases needs further investigations, the above results suggest that it may be a potential pharmacologic target in the treatment of pathological hypertrophy associated with different cardiac stress contexts.

## 5. ERK Activity in Mice Overexpressing RTKs in Cardiomyocytes

The role of ERK downstream to RTKs signaling in maladaptive cardiac hypertrophy has been scantly studied. RTK signaling is essential for normal human cardiac function, and the genetic deletion of RTKs can cause dilated cardiomyopathies [80,96,97,98]. ErbB2 conditional deletion models develop heart failure and isolated cardiomyocytes from these mice are more sensitive to doxo [80]. ErbB2 overexpression in the heart activates protective signaling pathways (PI3K, mTOR, and Bcl-2) and induces hypertrophy without heart failure [99]. A cardiomyocyte-specific constitutively active fibroblast growth factor (FGF) receptor mouse model showed concentric hypertrophy with increased cardiac mass and cardiomyocyte size, interstitial fibrosis, and myocyte disarray characteristic of hypertrophic cardiomyopathy, without consistent ERK activation [100]. We demonstrated that the constitutive activation of the Met proto-oncogene, the receptor for hepatocyte growth factor (HGFR), leads to the development of concentric cardiac hypertrophy [101]. The overactivation of Met produces a strong induction of ERK phosphorylation. Interestingly, we demonstrated that the initially formed concentric hypertrophy is adaptive and allows for the maintenance of the contractile force. At a later stage, the hypertrophic remodeling converges into heart failure [101]. Importantly, the pro-hypertrophic effects mediated by overactivated Met receptor were attenuated by the treatment with pimasertib, an MEK1 inhibitor. The attenuation of ERK signaling led to the prevention of heart failure progression [101]. On the other hand, another transgenic mouse with cardiac specific overexpression of HGF did not develop cardiac hypertrophy, indicating negative regulatory mechanisms on ligand-activated Met receptor signaling [102]. These data suggest that the level and the duration of the ERK pathway is crucial to define the pro-hypertrophic response. The EGFR was also associated with hypertrophic development in response to AngII detrimental stimulus [103,104]. Data showed that AngII induces the ERK pathway by c-Src-mediated transactivation of EGFR. The pathological hypertrophy induced by AngII was attenuated by the oral administration with EGFR inhibitors [104]. Finally, the ERK cascade is involved in a norepinephrine-induced pro-hypertrophic process via stimulation of IGF1R (insulin-like growth factor 1 receptor) [68,105,106]. Although the IGF1/PI3K/Akt pathway usually results in physiological hypertrophy, sustained Akt stimulation may induce pathological growth. The role of ERK in the Akt-dependent detrimental function has yet to be elucidated. Overall, these data suggest that fine tuning of RTK signaling could be a functional therapy to treat the maladaptive hypertrophic process.

## 6. ERK in Genetic Diseases with Hypertrophy

A significant percentage of cardiomyopathies have a genetic base. The genes involved in these disorders have been increasingly discovered. Diseases with a similar phenotype are characterized by mutations on genes encoding proteins belonging to the same pathway or with a similar structure and function. Hypertrophy is the main feature of the hypertrophic cardiomyopathies (HCMs) and RASopathy disorders. In this section, we elucidate the role of ERK cascade in the hypertrophic process involved in genetic hypertrophic cardiomyopathies.

### 6.1. Hypertrophic Cardiomyopathies (HCMs)

HCMs are inherited diseases characterized by genetic abnormalities in contractile proteins, also known as “disease of the sarcomere.” The majority of mutations characterizing HCMs are single nucleotide substitutions. The mutated proteins are incorporated in the sarcomere, where they produce non-functional contractile structure [107]. These genetic diseases are the most frequently occurring cardiomyopathies and are the main cause of cardiac sudden death in young people. The two most involved genes are β myosin heavy chain (βMHC), which is also the first identified mutation, and myosin-binding protein C. The other mutated genes so far identified in HCMs are cardiac troponin T (cTnT) and I (cTnI), myosin light chains, titin, α-tropomyosin, α-actin, α-myosin heavy chain, and muscle LIM protein [108]. Furthermore, mutations in proteins involved in the regulation of cardiac metabolism were found associated with HCMs: γ-2-regulatory subunit of the AMP-activated protein kinase (PRKAG2) and the lysosome-associated membrane protein 2 (LAMP-2). In addition, the mitochondrial myopathies due to mutations in mitochondrial DNA (including Kearns-Sayre syndrome) or mitochondrial proteins are classified as HCM diseases [109]. Surely, many other mutations leading to HCMs have yet to be identified. Importantly, HCMs are associated with activation of the ERK cascade [110,111]. Guinea pig cardiomyocytes infected with adenovirus expressing human HCM mutations in cTnT, cTnI, and α tropomyosin (αTM) showed increased myofilament Ca^2+^ sensitivity—resulting in hypertrophy and contractile dysfunction—and overstimulation of ERK [111]. ERK was also super-induced in a model of βMHC Q (403) mutation. The reduction of ERK activity in these transgenic rabbits led to improved cardiac contractility through regression of hypertrophy and fibrosis [110]. Recently, Davis and colleagues [112] demonstrated that ERK is involved in the directionality of cardiac growth by mediating the addition of sarcomeres in parallel to thicken cardiomyocytes. Isolated adult rat cardiomyocytes transduced with I61Q cardiac troponin C (cTnC) adenovirus and subjected to 48 h of chronic pacing showed failure of ERK translocation to the nucleus, compared to wild-type cells. The retention of ERK in the cytosol led to increased myocyte length, which correlated to dilated growth through cardiac cells elongation. Importantly, double transgenic mice expressing activated MEK1 and the I61Q cTnC mutation rescued the cardiac function and presented concentric remodeling and myocyte thickening [112]. Moreover, R193H cTnI mutation caused a significant translocation of ERK to the nucleus, leading to reduced cardiomyocyte length and increased width both at baseline and after chronic pacing. Co-transduction of R193H cTnI and dominant-negative MEK1 adenoviruses blocked ERK translocation and reduced the concentric phenotype [112]. These data indicate that mutations on sarcomeric proteins can influence the cellular location of ERK, which determinates the type of cardiac growth in response to stressed states. Further investigation is necessary to better elucidate the molecular mechanisms involving ERK in HCMs. The discovery of ERK-associated scaffolding proteins linked to the sarcomeres might shed light in these mechanisms.

### 6.2. RASopathies

RASopathies are human developmental genetic syndromes characterized by germline mutations in components of the RAS-ERK cascade. The first genes identified to be mutated in RAS-related syndromes were neurofibromin 1 and protein tyrosine phosphatase, nonreceptor type 11 (PTPN11) (which encodes SHP2). These discoveries suggested the important roles mediated by RAS cascade in development. The causative germline mutations are dominant gain-of-function, except for PTPN11 mutation, which is dominant-negative [8]. RASopathies are phenotypically similar diseases comprising Noonan syndrome, Costello syndrome, Cardio-facio-cutaneous syndrome, and Leopard syndrome. The common phenotype includes short stature, mental retardation, craniofacial dysmorphology, and heart defects such as aortic stenosis, septal defects, and mitral insufficiency. A subset of these syndromes often manifests cardiac hypertrophy similar to HCMs. Indeed, Costello and Leopard syndromes show—with high frequency—hypertrophy, instead of Cardio-facio-cutaneous and Noonan syndromes, which develop HCMs less frequently [8,113]. Importantly, experimental data suggest the role of ERK in cardiomyocyte hypertrophy of RASopathies. In fact, muscle-specific deletion of PTPN11 produces dilated cardiomyopathy in the absence of the hypertrophic process. The knockout cardiomyocytes showed a strong reduction in ERK activity in response to stress stimuli, such as pressure overload. These data suggest that PTPN11 is essential for the induction of the adaptive hypertrophic response by regulation of ERK cascade [114]. Furthermore, the ectopically expression of HCM-associated RAF-1 mutations from Noonan and Leopard patients showed enhancement in ERK activity with respect to non–HCM-associated mutants [115]. Mechanistically, mutated RAF-1 protein presents reduced phosphorylation at serine 259, an inhibitory phosphorylation site, resulting in dissociation from the scaffold protein 14-3-3 and induction in ERK activation [116]. Since KSR interacts with 14-3-3, the involvement of ERK-associated scaffolding proteins remains to be clarified in RASopathies. Thus, RASopathies demonstrate the importance of the signaling intensity in the development of non-functional patterns in heart physiology. In addition, knockin mice expressing the Noonan syndrome-associated RAF-1 (L613V) mutation showed eccentric cardiac hypertrophy and heart failure following pressure overload. Importantly, MEK inhibition normalized the cardiac defects in mutated mice, indicating that the enhancement in ERK activity causes HCMs and that HCMs require a mutation-specific therapeutic approach [117]. HCMs developed in RAS-related syndromes have a similar hypertrophic phenotype with respect to non-syndromic HCMs. Thus, the therapeutic strategies identified for RASopathies may also be exploitable for non-syndromic HCMs.

## 7. Targeting ERK to Therapeutically Modulate the Cardiac Hypertrophy

Treatments targeting the hypertrophic process aim to delay or even reverse the maladaptive remodeling. To achieve this goal, the molecular mechanism(s) and signaling pathway(s) involved in the hypertrophic process have to be highlighted showing the similarities and differences that distinguish pathological from physiological hypertrophy. Although ERK activation events require further dissection and refinements, ERK or its MAPKKs are possible therapeutic targets for a pharmacological strategy against cardiac hypertrophic diseases. Kinase inhibitors newly developed or already in use in the oncological field, could be exploited to mitigate excessive signaling activation in cardiac hypertrophy. We demonstrated that pharmacological inhibition of ERK activation with Pimasertib, an orally bio-available small-molecule inhibitor of MEK1/2, mitigates the cardiac hypertrophic phenotype of mice expressing the constitutively activated form of Met tyrosine kinase receptor in the heart. The mitigation and therefore the normalization of the ERK activity level were beneficial without exerting detrimental effects [101]. In line with this, Li and colleagues showed that another drug targeting the ERK pathway, selumetinib, could attenuate pressure overload-induced cardiac hypertrophy in vivo. Selumetinib treatment prevented cardiomyocyte enlargement, fetal gene overexpression, and cardiac fibrosis, which are all hallmarks of pathological hypertrophy [118]. Furthermore, ERK cascade inhibitors were tested as potential therapeutic agents for the treatment of the RASopathies disorders. Chronic MEK-ERK activity is critical for causing HCMs in Noonan syndrome. Indeed, Wu and colleagues showed that postnatal MEK inhibitor treatment (PD0325901) of the knockin mouse model for syndrome-associated RAF-1 (L613V) mutation effectively normalized cardiac anatomy and function [117]. Altogether, these preclinical studies are encouraging towards the use of the MEK-ERK pathway inhibitors as safe and effective cardiovascular drugs targeting cardiac hypertrophy with aberrant ERK activation. However, considering that the cardiac hypertrophy differently affects each patient, a standard plan of treatment cannot be designed, and an individualized therapy is required. This approach of “precision medicine” could be important for the public health since efficacious therapeutic strategies directly interfering with the hypertrophy development are still lacking. In fact, the management of symptomatic cardiac hypertrophy is based on medications relieving symptoms or, at worst, surgical procedures. However, a word of caution is warranted regarding the use and development of ERK-targeted therapies, as the ERK cascade is well known to exert cytoprotective functions. In particular, the total blockage of ERK cascade during cardiac stress, such as pressure overload, MI, and oxidative stress, could produce important irreversible cardiac damage. The development of new selective inhibitors leaving the ERK antiapoptotic effects intact may pave the way in future to impairment of ERK-driven pathological hypertrophy.

## 8. Conclusions

Cardiac hypertrophy is a complex response to various physiological and pathological stimuli. Adaptive concentric hypertrophy is a compensatory process that counteracts the hemodynamic overload. When the pathological stimulus is sustained and chronic, hypertrophy becomes eccentric and maladaptive, leading to heart failure. There is no doubt that the ERK cascade is a key player in the hypertrophic process. Activation of the ERK pathway is involved in both adaptive and maladaptive hypertrophy, depending on the pathophysiological context (Table 1).

However, some issues remain controversial and need to be elucidated. In particular, ERK seems to exert beneficial hypertrophic effects during chronic pressure overload, through cell death prevention and regulation of hypertrophy. On the other hand, ERK participates in detrimental hypertrophy during hypertension and cardiac side effects mediated by chemotherapy. It appears that not all ERK activation events are the same. Duration, intensity, frequency, and cytoplasmic versus nuclear localization of activated ERK may influence the final outcome. β-arrestin recruitment to GPCRs induces selective coupling to ERK beneficial activation. ERK-associated scaffold proteins are critical determinants for the spatial specificity of the signal. IQGAP1 and Melusin are involved in the adaptive pro-hypertrophic process, conversely FHL1 and ANKRD1 are associated with maladaptive hypertrophy. In addition, ERK phosphorylated at threonine 188 and ERK5 isoform are associated with pathological hypertrophy. ERK activation is found in the hypertrophic cardiomyopathy of sarcomere genetic diseases and in RASopathies. These findings strengthen the view that ERK is important for hypertrophy. Overall, these data suggest that both the activation and the inhibition of the ERK cascade could be targeted for therapy in cardiac hypertrophy. Given the importance of the RAS-RAF-MEK-ERK pathway in cancer, novel pharmacological ERK inhibitors will be available in the near future that could be of interest for their clinical applicability in cardiac disease. In parallel, the use of ERK-targeted therapies in cancer must be deeply evaluated to avoid unwanted side effects in the heart.

## Figures and Tables

**Table 1:** Modulation of ERK signaling by extrinsic and intrinsic stimuli.

Extrinsic Stimuli			
	Experimental Models	Response	References
Pressure Overload	Transverse aortic constriction (TAC) in mice	Early adaptive concentric hypertrophy; ERK ↑; Late detrimental eccentric hypertrophy; ERK ↓	[19]
Aortic Valve Stenosis	Human patients	Detrimental eccentric hypertrophy; ERK ↓	[87]
AngII	Cardiomyocytes	ERK5 ↑; ERK ↑	[10,93,104]
AngII inhibitors	Heart failure patients	Reduction of cardiac hypertrophy and heart failure; ERK ↓	[24]
Isopreteronol	βAR stimulation in mice	Cardiac hypertrophy and fibrosis; ERK phosphorylation at T188 ↑	[88]
βAR Blockers	In vitro treatment	Arrestin-mediated EGF receptor transactivation; ERK ↑	[28,29]
Anthracycline	In vitro and in vivo treatments	Heart failure; ERK ↑	[71,72]
	In vitro and in vivo treatments	Cardioprotective action; ERK ↑	[74,75,76]
	Rat cardiomyocytes (Lapatinib and Doxorubicin)	Cardiotoxicity; ERK ↓	[84]
Trastuzumab	Human cardiomyocytes	Cardiotoxicity; ERK ↑	[85]
**Intrinsic Stimuli**			
	**Experimental Models**	**Response**	**References**
DN RAF-1	Cardiomyocytes-specific Tg mice	Blunted response to pathological hypertrophy; ERK ↓	[30]
DUSP-6	Cardiomyocytes-specific Tg mice	Heart failure in response to TAC; ERK ↓	[32]
MEK1	Cardiomyocytes-specific Tg mice	Concentric cardiac hypertrophy; ERK ↑	[4]
MEK5β	Cardiomyocytes-specific Tg mice	Eccentric cardiac hypertrophy and heart failure; ERK5 ↑	[10]
MEK5α	Cardiomyocytes-specific Tg mice	Prevention of heart failure in response to MI; ERK5 ↑	[94]
ERK5	Cardiomyocytes-specific knock out mice	Reduced cardiac hypertrophy, and increased apoptosis in response to TAC; ERK5 ↓	[91]
αAR	α(1A/C)AR and α(1B)AR double knock out mice	Small heart with reduced cardiac output in response to TAC; ERK ↓	[34]
α1AR	knock out mice	Pathological hypertrophy and heart failure in response to MI; ERK ↓	[35]
β1AR	Cardiomyocytes-specific Tg mutant mice	Lack of EGFR transactivation; Increased contractility, fibrosis and apoptosis; ERK ↓	[28]
βArrestin	In vitro knock out	Arrestin 1: ERK ↑; Arrestin 2: ERK ↓	[21]
βArrestin	Knock out mice	Lack of EGFR transactivation; ERK ↓	[28,29]
Erbin	Knock out mice	Cardiac hypertrophy and heart failure in response to TAC; ERK ↓	[42]
IQGAP1	Knock out mice	Eccentric hypertrophy in response to TAC; ERK ↓	[44]
Melusin	Cardiomyocytes-specific Tg mice	Concentric hypertrophy, improved response to TAC; ERK ↑	[47]
FHL1	Knock out mice	Blunted response to pathological hypertrophy; ERK ↓	[48]
ANKRD1	Cardiomyocytes knock down and knock out mice	Blunted response to pathological hypertrophy; ERK ↓	[51]
ERK2 T188A	Cardiomyocytes-specific Tg mice	Attenuation of pathological hypertrophy in response to GPCRs activation and TAC	[87,88]
HGFR	Cardiomyocytes-specific Tg mice	Early adaptive concentric hypertrophy; late heart failure; ERK ↑	[101]
EGFR	In vitro and in vivo protein knock down	Failure of AngII-mediated cardiac hypertrophy; ERK ↓	[103,104]
IGF1R	Cardiomyocytes-specific protein knock down in mice	Attenuation of norepinephrine-induced cardiac hypertrophy; ERK ↓	[105]
HCM	βMHC-Q(403) in Tg rabbits	Cardiac hypertrophy, fibrosis, and contractile dysfunction; ERK ↑	[110]
	cTnT R92Q, cTnI R145G, and αTM D175N in cardiomyocytes	Cardiomyocyte hypertrophy; ERK ↑	[111]
	I61Q cTnC in cardiomyocytes	Failure of ERK translocation to the nucleus and cardiomyocytes elongation	[112]
	R193H cTnI in cardiomyocytes	ERK translocation to the nucleus and increased cardiomyocytes width	[112]
RASopathies	Cardiomyocytes-specific knock out of PTPN11 in mice	Failure in the induction of adaptive hypertrophy; ERK ↓	[114]
	Noonan RAF-1 L613V mutation knock in mice	Eccentric hypertrophy and heart failure; ERK ↑	[117]

↑ increased or ↓ decreased levels of phosphorylation at regulatory TEY site.

## Abbreviations

αAR	α-adrenergic receptor
αTM	α tropomyosin
AngII	angiotensin II
ANKRD1	ankyrin repeat domain 1
βAR	β-adrenergic receptor
βMHC	β myosin heavy chain
BMK1	big MAPK 1
CTX	cardiotoxicity
cTnC	cardiac troponin C
cTnI	cardiac troponin I
cTnT	cardiac troponin T
Doxo	doxorubicin
ECM	extracellular matrix
EGFR	epidermal growth factor receptor
ErbB2	erythroblastic leukemia viral oncogene homolog 2
ERK	extracellular signal-regulated kinase
FGF	fibroblast growth factor
FHL1	four and a half LIM domain protein-1
GPCR	G-protein-coupled receptor
GSK3	glycogen synthase kinase III
HCM	hypertrophic cardiomyopathy
HER2	epidermal growth factor receptor 2
HGFR	hepatocyte growth factor receptor
HSF	heat shock factor
IGFR	insulin-like growth factor receptor
IQGAP1	IQ motif-containing GTPase-activating protein 1
LAMP-2	lysosome-associated membrane protein 2
MAPK	mitogen-activated protein kinase
MI	myocardial infarction
NF-κB	nuclear factor kappa B
PRKAG2	γ-2-regulatory subunit of the AMP-activated protein kinase
PTPN11	protein tyrosine phosphatase, nonreceptor type 11
ROS	reactive oxygen species
RTK	receptor tyrosine kinase
TAC	transverse aortic constriction
T188	threonine 188
Tg	transgenic mice
